# Genome-Wide Association Mapping Identifies Key Genomic Regions for Grain Zinc and Iron Biofortification in Bread Wheat

**DOI:** 10.3389/fpls.2022.903819

**Published:** 2022-06-30

**Authors:** Philomin Juliana, Velu Govindan, Leonardo Crespo-Herrera, Suchismita Mondal, Julio Huerta-Espino, Sandesh Shrestha, Jesse Poland, Ravi P. Singh

**Affiliations:** ^1^Borlaug Institute for South Asia, Ludhiana, India; ^2^International Maize and Wheat Improvement Center, Texcoco, Mexico; ^3^Campo Experimental Valle de Mexico, Instituto Nacional de Investigaciones Forestales, Agricolas y Pecuarias, Chapingo, Mexico; ^4^Department of Plant Pathology, Wheat Genetics Resource Center, Kansas State University, Manhattan, KS, United States; ^5^Biological and Environmental Science and Engineering Division, King Abdullah University of Science and Technology, Thuwal, Saudi Arabia

**Keywords:** GWAS, wheat, zinc, iron, biofortification, X-ray fluorescence (XRF), RefSeq

## Abstract

Accelerating breeding efforts for developing biofortified bread wheat varieties necessitates understanding the genetic control of grain zinc concentration (GZnC) and grain iron concentration (GFeC). Hence, the major objective of this study was to perform genome-wide association mapping to identify consistently significant genotyping-by-sequencing markers associated with GZnC and GFeC using a large panel of 5,585 breeding lines from the International Maize and Wheat Improvement Center. These lines were grown between 2018 and 2021 in an optimally irrigated environment at Obregon, Mexico, while some of them were also grown in a water-limiting drought-stressed environment and a space-limiting small plot environment and evaluated for GZnC and GFeC. The lines showed a large and continuous variation for GZnC ranging from 27 to 74.5 ppm and GFeC ranging from 27 to 53.4 ppm. We performed 742,113 marker-traits association tests in 73 datasets and identified 141 markers consistently associated with GZnC and GFeC in three or more datasets, which were located on all wheat chromosomes except 3A and 7D. Among them, 29 markers were associated with both GZnC and GFeC, indicating a shared genetic basis for these micronutrients and the possibility of simultaneously improving both. In addition, several significant GZnC and GFeC associated markers were common across the irrigated, water-limiting drought-stressed, and space-limiting small plots environments, thereby indicating the feasibility of indirect selection for these micronutrients in either of these environments. Moreover, the many significant markers identified had minor effects on GZnC and GFeC, suggesting a quantitative genetic control of these traits. Our findings provide important insights into the complex genetic basis of GZnC and GFeC in bread wheat while implying limited prospects for marker-assisted selection and the need for using genomic selection.

## Introduction

Malnutrition, an incessant threat to the sustainability and resilience of healthy food systems has been exacerbated by the COVID-19 pandemic (Carducci et al., 2021). Currently, the world is grappling with an alarming increase in the number of undernourished people which was estimated to be about 768 million in 2020, with Asia and Africa being the biggest contributors (FAO et al., 2021). Of particular concern are the staggering numbers of malnourished children and in 2020, it was estimated that 149.2 million children under 5 years of age were stunted, 45.4 million were wasted and 38.9 million were overweight (WHO, 2021). Additionally, “micronutrient malnutrition” that refers to the inadequate intake of vitamins and minerals (zinc, iodine, iron, etc.) essential for the proper growth and development of the body poses a serious threat to both children and pregnant women living in developing countries with low and middle income (Welch and Graham, 2000; Pfeiffer and McClafferty, 2008; HLPE, 2020; WHO, 2020).

Zinc is a key micronutrient, whose deficiency results in increased early childhood mortality, morbidity, and stunting, impairs mental development, and aggravates susceptibility to diseases like diarrhea, malaria, and pneumonia (Hotz and Brown, 2004; Black et al., 2008; Ackland and Michalczyk, 2016). Globally, an estimated 17.3% of the population is fraught with the risk of inadequate zinc intake (Wessells and Brown, 2012), and its deficiency is widely prevalent in developing countries like India (Stein et al., 2007). Iron is another essential micronutrient, whose deficiency impairs cognitive development and physical activity, results in increased mortality rates primarily in children and women, and is a predominant cause of anemia (Stein et al., 2008; Safiri et al., 2021). In 2019, an estimated 29.9% of the women between 15 and 49 years were affected by anemia, which is widespread in Central and Southern Asia and sub-Saharan Africa (FAO et al., 2021; WHO, 2021).

To mitigate micronutrient deficiencies and the disease burden arising from them, several countries have adopted interventions like industrial food fortification and pharmaceutical supplementation. However, these interventions have not been entirely successful because of their inability to reach rural people and the urban poor who do not consume a lot of processed food and the high costs involved in producing and buying fortified food that developing countries cannot afford (Stein et al., 2007, 2008). Hence, “biofortification,” which refers to the breeding of staple crops for higher micronutrient concentrations, has emerged to be a sustainable and cost-effective strategy to combat the micronutrient deficiency challenge concomitantly with other interventions. Biofortified crops have the potential to target low-income households and become part of the food chain even in rural areas where people do not have access to commercially fortified food and heavily consume local food staples (Bouis, 2002; Nestel et al., 2006; Stein et al., 2007, 2008; Pfeiffer and McClafferty, 2008; Bouis and Welch, 2010; Bouis et al., 2011).

Cereal-based foods contribute substantially to the daily diet in countries where micronutrient deficiencies are prevalent (Bouis and Welch, 2010). Wheat, a major cereal that provides 20% of the dietary calories and proteins worldwide (Shiferaw et al., 2013), has been an ideal target for biofortification, as biofortified wheat can significantly ameliorate the consequences of micronutrient deficiencies (Srinivasa et al., 2014; Sazawal et al., 2018). While agronomic biofortification of wheat *via* soil and foliar application of zinc and iron fertilizers is an option (Cakmak, 2008; Cakmak et al., 2010; Aciksoz et al., 2011; Zou et al., 2012), it is not commonly used. Therefore, genetic biofortification that involves the application of both classical and novel molecular breeding approaches for characterizing and exploiting the genetic variability for grain zinc concentration (GZnC) and grain iron concentration (GFeC) remains to be a viable strategy to develop biofortified wheat (Velu et al., 2012, 2014). While the genetic variation for micronutrient concentration in cultivated wheat is narrow (Monasterio and Graham, 2000; Zhao et al., 2009), high micronutrient concentrations have been reported in wild relatives of cultivated wheat including *Triticum turgidum* ssp. *dicoccoides*, *T. spelta*, *T. monococcum*, *T. boeticum*, *T. polonicum*, *Aegilops kotschyi*, and *A. tauschii*, landraces, and synthetic hexaploid wheat (Cakmak et al., 2000, 2004; Monasterio and Graham, 2000; Chhuneja et al., 2006; Gomez-Becerra et al., 2010; Velu et al., 2011, 2019; Suchowilska et al., 2012). Hence, the biofortification program at the International Maize and Wheat Improvement Center (CIMMYT) supported by the HarvestPlus program and more recently by the “Accelerating the Mainstreaming of Elevated Zinc in Global Wheat Breeding” project breeds for biofortified bread wheat varieties by initially crossing micronutrient-rich genetic resources with high-yielding elite cultivated wheat lines. It has successfully developed and disseminated high-yielding zinc and iron biofortified wheat varieties with good resilience to biotic and abiotic stresses, end-use quality, and farmer preferred agronomic traits in South Asia (Ortiz-Monasterio et al., 2007; Velu et al., 2012, 2014, 2015, 2019; Guzmán et al., 2014).

Accelerating breeding efforts for biofortification of bread wheat with grain zinc and iron necessitates understanding their genetic control and identifying closely linked molecular markers for deployment in marker-assisted selection to select lines with favorable alleles (Xu et al., 2012; Velu et al., 2014; Tiwari et al., 2016). Several quantitative trait loci (QTL) mapping studies have dissected the genetic architecture of GZnC and GFeC in biparental populations and reported associated markers (Shi et al., 2008; Xu et al., 2012; Hao et al., 2014; Srinivasa et al., 2014; Crespo-Herrera et al., 2016, 2017; Tiwari et al., 2016; Velu et al., 2017c; Liu et al., 2019; Krishnappa et al., 2021; Rathan et al., 2021). However, QTL mapping studies can only identify the alleles segregating between the parents for the traits and involve significant population development time (Brachi et al., 2011; Korte and Farlow, 2013). Hence, genome-wide association mapping is a valuable alternative approach that can be used for identifying GZnC and GFeC associated markers as it does not require developing mapping populations, allows the use of any available population with diversity for the trait, and harnesses population-level linkage disequilibrium (LD) between markers and causal polymorphisms (Risch and Merikangas, 1996; Remington et al., 2001; Flint-Garcia et al., 2003; Yu and Buckler, 2006; Slatkin, 2008).

Given that only a few genome-wide association studies for GZnC and GFeC in bread wheat have been reported (Guttieri et al., 2015; Alomari et al., 2018; Bhatta et al., 2018; Velu et al., 2018; Tong et al., 2022), our primary objective was to perform genome-wide association mapping for GZnC and GFeC using a large panel of 5,585 elite breeding lines from CIMMYT’s bread wheat (referred to as BW) improvement program and zinc (referred to as ZN) improvement program. The BW improvement program mainly focuses on developing high-yielding varieties along with stress-resilience, whereas the ZN improvement program focuses on developing high GZnC varieties along with high grain yield, high GFeC, and stress-resilience. While most lines were grown in an optimally irrigated environment, we also grew some lines in a water-limiting drought-stressed environment and a space-limiting small plots environment to understand if common marker-trait associations could be identified across these environments. Moreover, we also aimed at identifying markers that were consistently associated with GZnC and GFeC in more than one dataset to understand the stability of the identified associations.

## Materials and Methods

### Populations and Environments for Grain Zinc and Iron Evaluation

For this study, we used the BW and ZN improvement program breeding lines that were developed using the selected-bulk breeding scheme (Singh et al., 1998). In this scheme, all the early-generation progenies were selected visually for agronomic traits, plant health, rust, etc., and then bulk harvested until the individual plants or head-rows derived small plots. The lines were grown at the Norman E. Borlaug Experiment Station, Ciudad Obregon (27°24′N, 109°56′W), Sonora, Mexico and were from the following nurseries and programs.

#### Parcelas Chicas or Small Plots From the Zinc Improvement Program

This nursery comprised progenies from the F_4_, F_5_, or F_6_ generations in the ZN improvement program, which were in the pre-yield testing stage and were planted in space-limiting small paired-rows plots or parcelas chicas (PCs) of 0.56 m^2^ for visual selection along with check varieties. The PCZN in each cycle comprise greater than 10,000 small plots from which a subset (about 1,100–1,600 small plots) selected visually for agronomic traits, plant health, grain characteristics, and rust resistance and for larger and plump grains after harvesting is evaluated for GZnC and GFeC. The PCZN lines that were grown in small plots during the 2017–2018 cycle (referred to by the harvest year as PCZN 2018 SP) were evaluated for GZnC, and the lines that were grown during the 2020–2021 cycle (PCZN 2021 SP) were evaluated for GZnC and GFeC.

#### Yield Trial Lines From the Bread Wheat Improvement Program and the Zinc Improvement Program

Selections from the pre-yield testing plots result in the yield trial (YT) nurseries in both the BW (YTBW) and zinc (YTZN) improvement programs. A subset of the 9,000 YTBW lines comprising about 1,100 lines and all the 1,100 YTZN lines were evaluated for GZnC and GFeC. Both the YTBW and the YTZN lines were grown in the bed-5 irrigations environment (B5IR), where the lines were planted on raised beds during the optimum planting time (late November to early December) and received optimum irrigation of 500 mm of water in total from five irrigations. An alpha-lattice design with two replications and each trial comprising six blocks and two high-yielding check varieties was used for the YT lines. The size of the YT plots was 4.8 m^2^, and the lines were sown in three rows over each of the two beds that were 80 cm wide. The grains of the YTBW lines and checks that were grown in the B5IR environment during the 2019–2020 cycle (YTBW 2020 B5IR) and the 2020–2021 cycle (YTBW 2021 B5IR) and the YTZN lines and checks that were grown during the 2018–2019 cycle (YTZN 2019 B5IR), 2019–2020 cycle (YTZN 2020 B5IR), and 2020–2021 cycle (YTZN 2021 B5IR) were evaluated for GZnC and GFeC.

#### Elite Yield Trial Lines From the Bread Wheat Improvement Program and the Zinc Improvement Program

Selections from the YT nurseries for grain yield and other traits result in the elite yield trial (EYT) nurseries in the BW (EYTBW) and the zinc (EYTZN) improvement programs, comprising about 1,100 lines and 250–300 lines, respectively, each year. While the trial design was similar to that in the YT nurseries, the EYTBW lines were grown in space-limiting small plots, the B5IR environment, and a water-limiting moderately drought-stressed environment, where the lines were planted during the optimum planting time in raised beds and received a total of about 200 mm of water in two irrigations (referred to as the bed-2 irrigations or the B2IR environment). The EYTBW lines grown in the B5IR environment, B2IR, and small plots during the 2020–2021 cycle (EYTBW 2021 B5IR, EYTBW 2021 B2IR, and EYTBW 2021 SP) and the EYTZN lines grown in the B5IR during the 2019–2020 cycle (EYTZN 2020 B5IR) and 2020–2021 cycle (EYTZN 2021 B5IR) were evaluated for GZnC and GFeC.

In all the environments, heterogeneity of zinc concentration in the soil was managed by the application of 25 kg ha^–1^ of ZnSO_4_ in every crop cycle (Velu et al., 2014, 2018).

### Grain Zinc and Iron Phenotyping

For all the environments and populations, when the grains were completely dry in the field after physiological maturity, the plots were harvested. About 20 g of grains from each genotype were sampled and cleaned from any impurities and broken grains. Cleaned grain samples from different environments were used for GZnC and GFeC analysis in a non-destructive bench-top energy-dispersive X-ray fluorescence spectrometry instrument (model X-Supreme 8000, Oxford Instruments, Abingdon, United Kingdom) that was standardized for high-throughput screening of these micronutrients in wheat grains (Paltridge et al., 2012). The GZnC and GFeC in parts per million (ppm) were obtained for one to three replications in different nurseries and environments as shown in Table 1, and outliers in the phenotypic data were removed.

**TABLE 1:** Populations, number of lines, number of markers, environments where the populations were grown, datasets for each population and environment, and the number of tests of association that were performed for grain zinc concentration and grain iron concentration.

Populations	Number of lines	Number of markers	Environments	Datasets	Number of tests of association
YTBW 2020	1,022	12,107	B5IR	Rep 1, Rep 2, BLUEs	72,642
EYTBW 2021			B5IR	Rep 1, Rep 2, BLUEs	72,642
			B2IR	Rep 1, Rep 2, BLUEs	72,642
			SP	Rep 1, Rep 2, BLUEs	72,642
			All	BLUEs	24,214
YTBW 2021	1,069	5,905	B5IR	Rep 1, Rep 2, BLUEs	35,430
YTZN 2019	1,091	6,413	B5IR	Rep 1, Rep 2, Rep3, BLUEs	51,304
PCZN 2018			SP	Rep 1 (only zinc)	6,413
YTZN 2020	278	11,496	B5IR	Rep 1, Rep 2, Rep3, BLUEs	91,968
EYTZN 2021			B5IR	Rep 1, Rep 2, BLUEs	68,976
YTZN 2021	539	5,703	B5IR	Rep 1, Rep 2, BLUEs	34,218
EYTZN 2020	241	12,047	B5IR	Rep 1, Rep 2, Rep3, BLUEs	96,376
PCZN 2021	1,589	8,988	SP	Rep 1	17,976
Combined panel	3,994	12,335	B5IR	Percentage check	24,670

*YTBW, yield trial bread wheat; EYTBW, elite yield trial bread wheat; YTZN, yield trial zinc; EYTZN, elite yield trial zinc; PCZN, parcela chica (small plots) zinc; B5IR, bed planting 5 irrigations; B2IR, bed planting 2 irrigations; SP, small plots; Rep 1, replication 1; Rep 2, replication 2; BLUEs, best linear unbiased estimates.*

Across replications, trials, and sub-blocks, the best linear unbiased estimates (BLUEs) for GZnC and GFeC in each of the populations and environments were calculated using the ASREML statistical package (Gilmour, 1997) with the following mixed model:


(1) $$y_{i\,j\,k\,l}=\mu +g_{i}+t_{j}+r_{k\,\left(j\right)}+b_{l\,\left(j\,k\right)}+\varepsilon_{i\,j\,k\,l}$$

where *y*_*ijkl*_ was the observed GZnC or GFeC, μ was the overall mean, *g*_*i*_was the fixed effect of the line, *t_j_* was the random effect of the trial that was independent and identically distributed $[t_{j}∼N\left(0,\sigma_{t}^{2}\right)$], *r*_*k*(*j*)_was the random effect of the replicate within the trial that was independent and identically distributed $\left[r_{k\,\left(j\right)}∼N\,\left(0,\sigma_{r}^{2}\right)\right]$, *b*_*l*(*jk*)_was the random effect of the incomplete block within the replicate and the trial that was independent and identically distributed $\left[b_{m\,\left(j\,k\right)}∼N\,\left(0,\sigma_{b}^{2}\right)\right],$ and ε_*ijkl*_ was the residual that was independent and identically distributed $\left[\varepsilon_{i\,j\,k\,l}∼N\,\left(0,\sigma_{\varepsilon }^{2}\right)\right]$. For EYTBW 2021, which was evaluated in three environments, we obtained BLUEs across environments by including the random effect of the environment in model (1), which is referred to as the EYTBW 2021 all environments BLUEs dataset. We also formed a combined panel with the BW and ZN lines that were grown in the B5IR environment in nurseries that had a common check Borlaug100, and we used the GZnC and GFeC expressed as percentages of the check Borlaug100 for GWAS.

### Genotyping

The genotyping-by-sequencing (GBS) approach (Poland and Rife, 2012; Glaubitz et al., 2014) was used to obtain genome-wide markers for all the populations. We used the TASSEL v5 (Trait Analysis by aSSociation Evolution and Linkage) GBS pipeline (Glaubitz et al., 2014) to call the marker polymorphisms and a minor allele frequency of 0.01 to discover marker single nucleotide polymorphisms. This was followed by anchoring 8,869,749 unique GBS tags using Bowtie2 (Langmead and Salzberg, 2012) to the first version of the reference sequence assembly of the bread wheat variety Chinese Spring (RefSeq version 1.0) (IWGSC, 2018) and the tags were named by their chromosomal location and physical position in RefSeq version 1.0. We then filtered the GBS tags using cutoffs for Fisher’s exact test values, inbred coefficients, and Chi-squared values as described in Juliana et al. (2019). The 102,619 marker polymorphisms that passed at least one of these filters were filtered further for missing data less than 50%, minor allele frequency greater than 5%, and heterozygosity less than 5%. Similarly, the lines with less than 50% missing genotyping data were removed, and we obtained 2,089 BW lines and 3,492 ZN lines (Table 1) that were used for analyses, along with checks. Missing marker data were imputed using the linkage disequilibrium-k-nearest neighbor genotype imputation method (Money et al., 2015) in TASSEL version 5 (Bradbury et al., 2007).

### Statistical Analysis of the Phenotypic Data, Marker Densities, and Population Structure Analysis

Statistical analysis of GZnC and GFeC BLUEs in the different datasets (Supplementary Table 1) was done and the mean, standard deviation, minimum, maximum, median, and range were obtained. Visualization of the GZnC and GFeC distributions within nurseries was done using the “R” package “ggplot2” (Wickham, 2009). To understand the impact of selecting for GZnC in the ZN improvement program, we used the GZnC in the B5IR environment expressed in percentage check Borlaug100 in YTZN 2020, YTZN 2021, and EYTZN 2021 and compared them with the corresponding BW improvement program nurseries, including YTBW 2020, YTBW 2021, and EYTBW 2021, that were not selected for GZnC. To make fair comparisons, we used all the lines in these nurseries instead of only the lines filtered for good genotyping data in Table 1. This included (i) 1,260 lines in YTZN 2020 (ii) 1,008 lines in YTZN 2021 (iii) 277 lines in EYTZN 2021 (iv) 1,500 lines each in YTBW 2020 and YTBW 2021 and (vi) 1,120 lines in EYTBW 2021.

The Pearson’s correlation between the GZnC and GFeC in different replications, environments within years, and environments across years were obtained, in addition to the correlations between GZnC and GFeC in different environments. The density of all the filtered markers in all the populations used in this study was assessed and the number of single nucleotide polymorphisms within a window size of 10 Mb was visualized using the “R” package “CMplot” (Lilin-yin, 2018). The first two principal components obtained in TASSEL v5 were then used to assess the population structure in the different nurseries (EYTBW, EYTZN, PCZN, YTBW, and YTZN) and in the combined panel (Price et al., 2006). Population structure was visualized using the “R” package “ggplot2” (Wickham, 2009) to understand the structure in the nurseries developed in different years and in the combined panel of BW and ZN lines.

### Genome-Wide Association Mapping for GZnC and GFeC

Genome-wide association mapping for GZnC and GFeC was done using all the 73 datasets described in Table 1 with the mixed linear model (Yu et al., 2006) using TASSEL version 5 (Bradbury et al., 2007). Population structure accounted for by using the first two principal components and kinship accounted for by the genomic relationship matrix using the centered identity-by-state method (Endelman and Jannink, 2012) were used as fixed and random effects, respectively, in the mixed linear model. We also used the optimum level of compression and the “population parameters previously determined” (Zhang et al., 2010) options for fitting the mixed linear model. The *p*-values for the tests of significance of the marker-trait associations were obtained along with the additive effects and percentage variation explained and the Manhattan plots with the −log_10_ *p*-values for GZnC and GFeC and the chromosomes were plotted using the “R” package “CMplot” (Lilin-yin, 2018). To correct for testing multiple hypotheses and to declare significant marker associations, we used the Bonferroni correction at an α level of 0.2. We also identified markers that were significantly associated with GZnC and GFeC in more than three datasets and visualized them on a reference map with their physical positions on the RefSeq version 1.0 using ‘‘Phenogram.’’^1^

## Results

### Phenotypic Data

**FIGURE 1:**
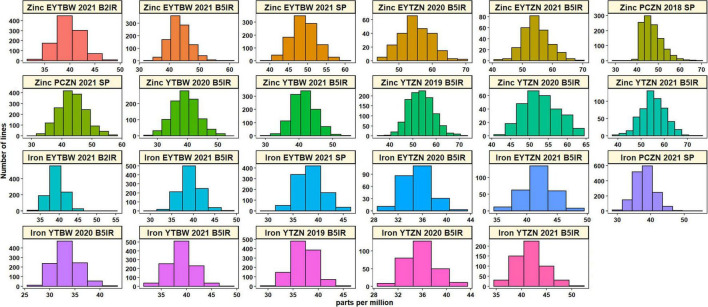
Distribution of the grain zinc concentration and grain iron concentration best linear unbiased estimates (BLUEs) in the yield trial bread wheat (YTBW), elite yield trial bread wheat (EYTBW), yield trial zinc (YTZN), and elite yield trial zinc (EYTZN) lines evaluated in the bed planting 5 irrigations environment (B5IR), bed planting 2 irrigations environment (B2IR), or small plots (SP), and the concentrations in the parcela chica (small plots) zinc (PCZN) lines.

**FIGURE 2:**
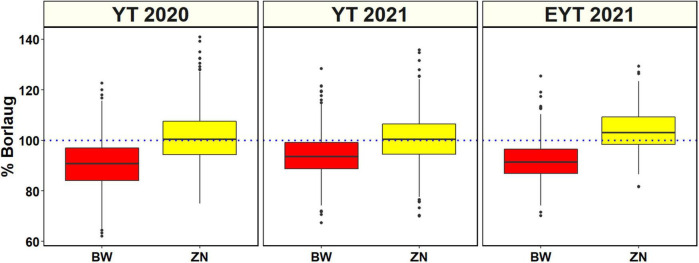
Distribution of the grain zinc concentration expressed as percentage check Borlaug100 values in the yield trial (YT) and elite yield trial (EYT) bread wheat (BW) and zinc (ZN) improvement program lines evaluated in the bed planting 5 irrigations environment during the 2020 and 2021 crop cycles.

Analysis of GZnC BLUEs (Supplementary Table 2 and Figure 1) indicated that the ZN lines from YTZN 2021 B5IR (55.2 ±5.4 ppm) and EYTZN 2020 B5IR (55.2 ± 4.7 ppm) had the highest mean GZnC followed by ZN lines from EYTZN 2021 B5IR (54.5 ± 4.5 ppm), YTZN 2019 B5IR (53.5 ± 5.3 ppm), and YTZN 2020 B5IR (52.9 ± 4.7 ppm). The highest GZnC of 74.7 ppm was observed in the ZN line MARASI #1 (GID9079797) from YTZN 2021. The mean GZnC in the ZN lines (51.7 ± 4.6 ppm) was higher than the mean GZnC in the BW lines (42.6 ± 3.9 ppm). Similarly, the mean GZnC expressed as percentage Borlaug100 was higher in the ZN nurseries including YTZN 2020 B5IR (101.2 ± 9.9%), YTZN 2021 B5IR (100.5 ± 9.2%), and EYTZN 2021 B5IR (104 ± 8.5%) compared to the corresponding BW nurseries including YTBW 2020 B5IR (90.7 ± 9.7%), YTBW 2021 B5IR (94 ± 7.7%), and EYTBW 2021 B5IR (91.9 ± 7.3%) (Figure 2). Moreover, we observed that the mean GZnC expressed as a percentage of Borlaug100 was 11.5, 6.7, and 13.2% higher in the ZN lines compared to the BW lines in YT 2020, YT 2021, and EYT 2021, respectively.

Analysis of GFeC BLUEs (Supplementary Table 2 and Figure 1) indicated that the ZN lines from EYTZN 2021 B5IR (41.9 ±2.4 ppm) and YTZN 2021 B5IR (41.8 ±2.8 ppm) had the highest mean GFeC, followed by the BW lines from EYTBW 2021 B5IR (39.3 ±2.4 ppm), EYTBW 2021 B2IR (39.3 ±2.2 ppm), and YTBW 2021 B5IR (38.9 ±2.4 ppm). The highest GFeC of 53.4 ppm was observed in the BW line KABUTA #1 (GID8776936), followed by the ZN line SHALIK #1 (GID9295875) with 52.7 ppm. The mean GFeC in the ZN lines (38.3 ±2.9 ppm) was similar to the BW lines (37.8 ± 2.6 ppm).

Among the EYTBW 2021 environments, we observed that the mean GZnC was high in the small plots environment, followed by the irrigated and drought-stressed environments. Similarly, the mean GFeC was high in the irrigated and drought-stressed environments, followed by the small plots environment in EYTBW 2021. The mean correlation across the replications was 0.56 ± 0.08 for GZnC and 0.44 ± 0.07 for GFeC (Supplementary Table 3). Across the irrigated, drought-stressed, and small plots environments in EYTBW 2021, the GZnC correlations ranged between 0.43 and 0.46, while the GFeC correlations ranged between 0.28 and 0.35. Considering the same lines that were analyzed in different years, we observed that the mean across-year correlations were 0.34 ± 0.13 and 0.42 ± 0.07 for GZnC and GFeC, respectively. Within the same year and environments, the mean correlation between GZnC and GFeC was 0.5 ± 0.09.

### Genotyping Data and Population Structure Analysis

**FIGURE 3:**
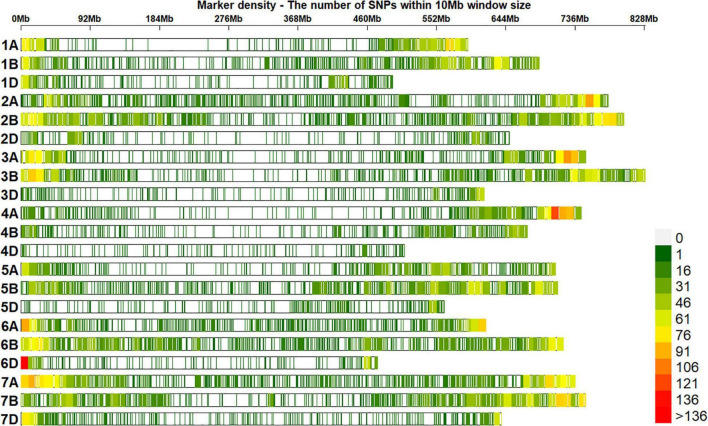
Densities of 20,184 genotyping-by-sequencing single nucleotide polymorphisms (SNPs) in the reference bread wheat genome (RefSeq v1.0). The color key with marker densities indicates the number of markers within a window size of 10 Mb.

**FIGURE 4:**
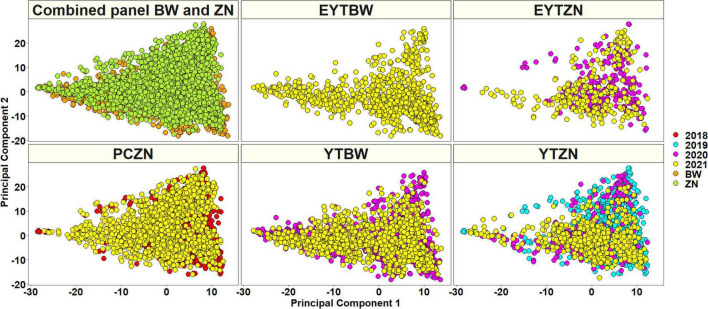
A plot of principal components 1 and 2 indicating the population structure in the (i) combined panel with the bread wheat (BW) and zinc (ZN) lines (ii) elite yield trial bread wheat (EYTBW) lines, (iii) elite yield trial zinc (EYTZN) lines, (iv) parcela chica (small plots) zinc (PCZN) lines, (v) yield trial bread wheat (YTBW) lines, and (vi) yield trial zinc (YTZN) lines. The colors in the combined panel indicate the lines from the BW and ZN improvement programs and the colors in all the other nurseries indicate the lines from different crop cycles (2018, 2019, 2020, and 2021).

A total of 20,556 unique GBS markers were used for genome-wide association mapping in different datasets. Considering the densities of the 20,184 markers with positions in the RefSeq version 1.0 within a window size of 10 Mb, we observed high densities in the telomeric ends and good marker coverage in all the chromosomes (Figure 3). We also observed that 38.2, 47.3, and 12.7% of the markers were on the A, B, and D genomes, while 1.8% of the markers were unaligned to any chromosome. Population structure analysis using the first two principal components indicated that the BW and ZN lines did not form clearly distinguishable clusters (Figure 4). Similarly, in the EYTBW, EYTZN, PCZN, YTBW, and YTZN nurseries, we did not observe distinguishable clusters of lines in the different crop cycles.

### Genome-Wide Association Mapping for Grain Zinc Concentration and Grain Iron Concentration

**FIGURE 5:**
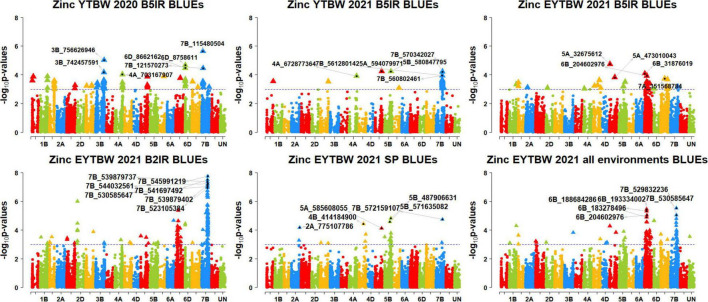
Manhattan plots showing the genomic regions significantly associated with grain zinc concentration in the bread wheat lines. BLUEs, best linear unbiased estimates; YTBW, yield trial bread wheat; EYTBW, elite yield trial bread wheat; B5IR, bed planting 5 irrigations; B2IR, bed planting 2 irrigations; SP, small plots.

**FIGURE 6:**
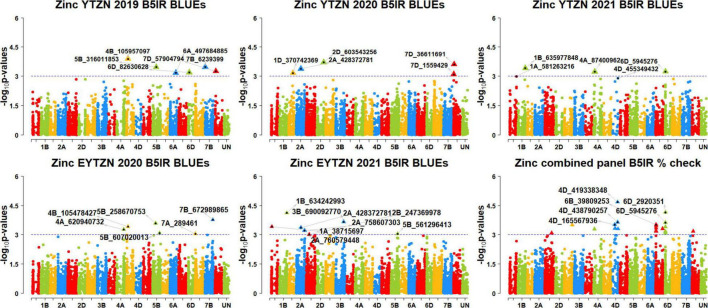
Manhattan plots showing the genomic regions significantly associated with grain zinc concentration in the zinc lines. BLUEs, best linear unbiased estimates; YTZN, yield trial zinc; EYTZN, elite yield trial zinc; PCZN, parcela chica zinc; B5IR, bed planting 5 irrigations; B2IR, bed planting 2 irrigations; SP, small plots.

**FIGURE 7:**
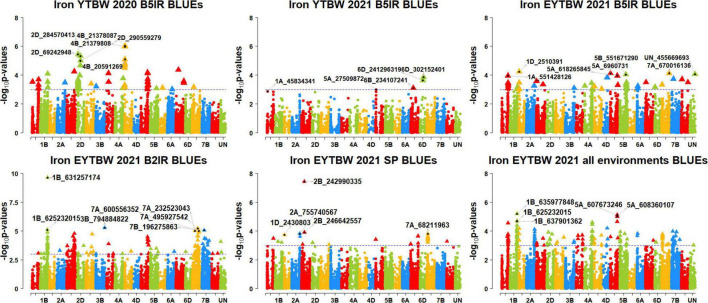
Manhattan plots showing the genomic regions significantly associated with grain iron concentration in the bread wheat improvement program lines. BLUEs, best linear unbiased estimates; YTBW, yield trial bread wheat; EYTBW, elite yield trial bread wheat; B5IR, bed planting 5 irrigations; B2IR, bed planting 2 irrigations; SP, small plots.

**FIGURE 8:**
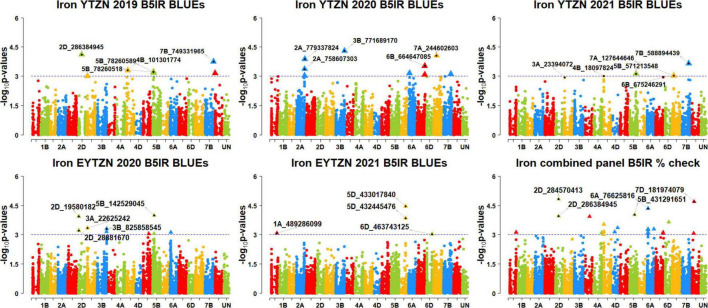
Manhattan plots showing the genomic regions significantly associated with grain iron concentration in the zinc improvement program lines. BLUEs, best linear unbiased estimates; YTZN, yield trial zinc; EYTZN, elite yield trial zinc; PCZN, parcela chica zinc; B5IR, bed planting 5 irrigations; B2IR, bed planting 2 irrigations; SP, small plots.

**FIGURE 9:**
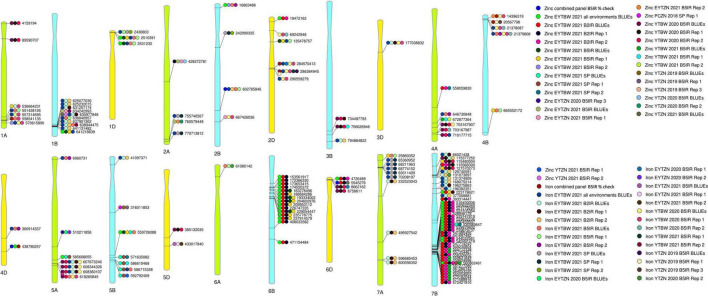
A reference map with 141-grain zinc concentration and grain iron concentration associated markers that were consistently associated in three to seven datasets, with physical positions in the reference sequence of bread wheat (RefSeq v 1.0). The datasets in which the markers were significantly associated include the replications 1 and 2 (Rep 1 and 2) and the best linear unbiased estimates (BLUEs) of grain zinc concentration and grain iron concentration in the yield trial bread wheat (YTBW), elite yield trial bread wheat (EYTBW), yield trial zinc (YTZN), elite yield trial zinc (EYTZN), and parcela chica zinc (PCZN) nurseries that were evaluated in the bed planting 5 irrigations (B5IR), bed planting 2 irrigations (B2IR), and small plots (SP) environments in different crop cycles.

We performed 742,113 association tests in 73 datasets and obtained the marker *p*-values, additive effects, and percentage variation explained. The genome-wide association mapping results were visualized using Manhattan plots showing the genomic regions significantly associated with GZnC and GFeC in different datasets (Figures 5–8). We identified 81 markers that were significant after Bonferroni correction for multiple testing at an α level of 0.2 (Supplementary Table 4). However, among these 81 markers significant after multiple testing corrections, none of them were significantly associated with GZnC and GFeC in more than one dataset. Hence, to avoid losing markers that were not significant after multiple testing and given the known complex genetic control of these traits, difficulties in phenotyping, and the effect of the environment on GZnC and GFeC, we considered all the markers that were associated with GZnC and GFeC in more than one dataset at a *p*-value threshold of 0.001 to be significant. So, among the 1,207 markers that were significantly associated with GZnC and GFeC at a *p*-value threshold of 0.001, only 427 markers were significantly associated in two or more datasets (Supplementary Table 4) and 141 markers in three or more datasets. These 141 markers were added to a reference map (Figure 9) and are highlighted below. In addition, we obtained the LD between the consistent markers using the standardized disequilibrium coefficient (D’) (Lewontin, 1964) and the correlations between alleles at the two marker loci (*r*^2^) with TASSEL version 5 and visualized them (Supplementary Data Sheet 2). Markers with high (>0.9) *r*^2^ and D’ values were considered as an LD block.

### Markers Significantly Associated With Grain Zinc Concentration Only

We observed that 67 markers were significantly associated with GZnC only in different datasets with the maximum additive effect being 1.7 ppm and the maximum percentage of phenotypic variation explained being 7.3% (Supplementary Tables 5, 6). Ten markers on chromosomes 1AS, 1BL, 2AL, 3BL, 4AL, 5DL, 6DS, and 7BS including 1A_4159194, 1A_89590707, 1B_634242993, 2A_760579448, 3B_756626946, 4A_672877364, 5D_385132035, 6D_8758611, 7B_115480504, and 7B_121570273 were associated with GZnC in irrigated environment datasets only. Seven markers on chromosome 7BL including 7B_393314447, 7B_541485456, 7B_541697492, 7B_547714243, 7B_549230804, 7B_558326652, and 7B_572421910 were associated with GZnC in drought-stressed environment datasets only. Marker 5B_571635082 was associated with GZnC in the small plots datasets only.

We also observed that 21 markers on chromosomes 2DS, 6B, 6DS, and 7BL including 2D_19472162, 6B_153561917, 6B_ 172366330, 6B_173653415, 6B_174550372, 6B_226747335, 6B_ 408033582, 6B_471154484, 6D_4726489, 7B_516450006, 7B_52 0632340, 7B_521279280, 7B_523105384, 7B_528946179, 7B_529 147819, 7B_529832236, 7B_530459507, 7B_539879402, 7B_5398 79737, 7B_544032561, and 7B_545991219 were associated with GZnC in the drought-stressed environment datasets and in the EYTBW 2021 all environments BLUEs dataset. Marker 5A_585608055 was associated with GZnC in the small plots datasets and in the EYTBW 2021 all environments BLUEs dataset.

Thirteen markers on chromosomes 2AL, 6DS, and 7B including 2A_428372781, 6D_5945276, 7B_119368300, 7B_555 712338, 7B_557354467, 7B_559631849, 7B_559924996, 7B_56 1277280, 7B_561280142, 7B_564053220, 7B_565054902, 7B_570 182044, and 7B_571588916 were associated with GZnC in the irrigated and drought-stressed environment datasets. In addition, eight markers on chromosome 6BS including 6B_183278496, 6B_188684286, 6B_193334002, 6B_204602976, 6B_209953112, 6B_229054447, 6B_235778775, and 6B_237914579 were associated with GZnC in the irrigated and drought-stressed environment datasets and in the EYTBW 2021 all environments BLUEs dataset.

Three markers on chromosome 5BL including 5B_316011853, 5B_586610468, and 5B_592792409 were associated with GZnC in the irrigated environment and small plots datasets. Marker 4A_558059830 on chromosome 4AL was associated with GZnC in the drought-stressed environment and small plots datasets. Marker 7B_530585647 on chromosome 7BL was associated with GZnC in the drought-stressed environment datasets, small plots datasets, and the EYTBW 2021 all environments BLUEs dataset. Marker 7B_560802461 was significantly associated with GZnC in the irrigated, drought-stressed and small plots datasets, in addition to the EYTBW 2021 all environments BLUEs dataset.

### Markers Significantly Associated With Grain Iron Concentration Only

We observed that 45 markers were significantly associated with GFeC only in different datasets with the maximum additive effect being 1.3 ppm and the maximum percentage of phenotypic variation explained being 8.3% (Supplementary Tables 7, 8). Among them, five markers on chromosomes 1AL, 2DS, 4BS, 5DL, and 6AS including 1A_558541135, 2D_69242948, 4B_20567798, 5D_433017840, and 6A_61080142 were associated with GFeC in irrigated environment datasets only. Four markers on chromosome 7A, including 7A_232523043, 7A_495927542, 7A_596685453, and 7A_600556352 were associated with GFeC in the drought-stressed environment datasets only. Marker 2B_242990335 on chromosome 2BS was associated with GFeC in the small plots datasets only.

Eleven markers on chromosomes 1AL, 1BL, 2DL, 4AL, 4BS, 4DL, and 5AS including 1A_551428126, 1A_557314695, 1B_636840957, 2D_284570413, 2D_286384945, 2D_290559279, 4A_646730848, 4B_21378087, 4B_21379808, 4D_360914337, and 5A_6960731 were associated with GFeC in irrigated environment datasets and in the EYTBW 2021 all environments BLUEs dataset. We also observed that 13 markers on chromosomes 1BL, 3BL, 5BS, and 7B including 1B_625077030, 1B_625232015, 1B_631257174, 1B_637901362, 1B_641131482, 3B_794884822, 5B_41097371, 7B_66021438, 7B_126740591, 7B_131374909, 7B_168075214, 7B_196275863, and 7B_377008881 were associated with GFeC in the drought-stressed environment datasets and in the EYTBW 2021 all environments BLUEs dataset.

Seven markers on chromosomes 2AL and 7AS including 2A_755740567, 2A_770713812, 7A_65360952, 7A_68211963, 7A_68774152, 7A_69311409, and 7A_70208197 were associated with GFeC in the small plots datasets and in the EYTBW 2021 all environments BLUEs dataset. Marker 7B_131073897 on chromosome 7BS was associated with GFeC in irrigated and drought-stressed environment datasets. Markers 1B_638944475 and 3D_177038832 on chromosomes 1BL and 3DS, respectively, were associated with GFeC in the irrigated and drought-stressed environment datasets and in the EYTBW 2021 all environments BLUEs dataset. Marker 1D_2430803 on chromosome 1DS was associated with GFeC in the irrigated and small plots environments and in the EYTBW 2021 all environments BLUEs dataset.

### Markers Significantly Associated With Grain Zinc Concentration and Grain Iron Concentration

We observed that 29 markers were associated with GZnC and GFeC in different datasets with the maximum additive effect being 1.8 ppm and the maximum percentage of phenotypic variation explained being 7% (Supplementary Tables 9, 10). Among them, marker 5A_608360107 was associated with seven datasets, and markers 1D_2510391, 5A_607673246, and 5A_608344326 were associated with six datasets. Marker 7B_223178621 on chromosome 7BS was associated with GZnC in an irrigated and drought-stressed environment dataset and in the EYTBW 2021 all environments BLUEs dataset, in addition to GFeC in a drought-stressed environment dataset. Marker 5B_559726088 on chromosome 5BL was associated with GZnC in irrigated and small plots environment datasets and in the EYTBW 2021 all environments BLUEs dataset, in addition to GFeC in an irrigated environment dataset. Marker 2B_16863488 on chromosome 2BS was associated with GZnC in an irrigated environment dataset and in the EYTBW 2021 all environments BLUEs dataset, in addition to GFeC in irrigated environment datasets. On chromosome 1DS, markers 1D_2510391 and 1D_2531230 were associated with GZnC and GFeC in irrigated environment datasets and in the EYTBW 2021 all environments BLUEs dataset. Marker 1B_641218839 on chromosome 1BL was associated with GZnC in irrigated environment datasets and with GZnC and GFeC in the EYTBW 2021 all environments BLUEs dataset.

Nine markers on chromosomes 2BL, 4AL, 4B, 5AL, 6DS, and 7AS including 2B_667426036, 4A_703167907, 4A_703167987, 4B_14396319, 4B_665552172, 5A_510211856, 5A_618265845, 6D_8662162, and 7A_25860352 were associated with GZnC and GFeC in irrigated environment datasets. Marker 1A_536664231 on chromosome 1AL was associated with GZnC and GFeC in irrigated environment datasets and GFeC in a drought-stressed environment dataset. Four markers on chromosomes 2BL and 5AL including 2B_602765846, 5A_607673246, 5A_608344326, and 5A_608360107 were associated with GZnC and GFeC in irrigated environment datasets and GFeC in drought-stressed environment datasets and in the EYTBW 2021 all environments BLUEs dataset.

Markers 1A_570615890 and 2D_135478757 on chromosomes 1AL and 2DS were associated with GZnC and GFeC in irrigated environment datasets and GFeC in the EYTBW 2021 all environments BLUEs dataset. Markers 3B_734497793 and 7B_115377252 on chromosomes 3BL and 7BS were associated with GZnC in irrigated environment datasets and GFeC in drought-stressed environment datasets. Markers 1B_635977848 and 7B_196382351 on chromosomes 1BL and 7BS were associated with GZnC in irrigated environment datasets and GFeC in drought-stressed environment datasets and in the EYTBW 2021 all environments BLUEs dataset. Marker 4A_710177715 on chromosome 4AL was associated with GZnC in irrigated environment datasets and with GFeC in the EYTBW 2021 all environments BLUEs dataset. Marker 5B_586715328 on chromosome 5BL was associated with GZnC in the small plots and with GFeC in an irrigated environment dataset and in the EYTBW 2021 all environments BLUEs dataset. Marker 4D_438790257 on chromosome 4DL was associated with GZnC in the EYTBW 2021 all environments BLUEs dataset and with GZnC and GFeC in the irrigated combined panel datasets.

## Discussion

In this study, we have analyzed the GZnC and GFeC in wheat breeding lines from CIMMYT’s BW and ZN improvement programs. Our results showed the existence of a large and continuous variation for GZnC and GFeC in the lines from both the programs, with the GZnC ranging from 27 to 74.5 ppm and GFeC ranging from 27 to 53.4 ppm. The nurseries from the ZN improvement program had higher mean GZnC compared to those from the BW improvement program, which is expected given that the ZN improvement program’s primary focus is breeding for higher GZnC by crossing with GZnC rich parents and the BW improvement program’s primary focus is maximizing grain yield (Guzmán et al., 2014). In addition, we have reported 6.7–13.2% higher GZnC in nurseries from the ZN improvement program compared to those from the BW improvement program, indicating significant progress from selecting for high GZnC in the ZN improvement program compared to the BW improvement program lines that were not selected for GZnC. The targeted breeding for high GZnC at CIMMYT has led to the release of more than 20 high zinc wheat varieties in target countries (Table 2) occupying more than 2.2 million households during the 2020–21 period.

**TABLE 2:** List of high zinc wheat varieties released globally.

S. No.	Name	Pedigree	Country	Year of release	Public/Private
1	Zinc Shakthi (Chitra)	CROC_1/AE.SQUARROSA (210)//INQALAB 91*2/KUKUNA/3/PBW343*2/KUKUNA	India	2014	Private/PVS
2	WB 02	T.DICOCCON CI9309/AE.SQUARROSA (409)//MUTUS/3/2*MUTUS	India	2016	Public
3	PBW-Zn01	T.DICOCCON CI9309/AE.SQUARROSA (409)/3/MILAN/S87230//BAV92/4/2*MILAN/S87230//BAV92	India	2016	Public
4	Ankur Shiva	T.DICOCCON CI9309/AE.SQUARROSA (409)/3/MILAN/S87230//BAV92/4/2*MILAN/S87230//BAV92	India	2018	Private
5	HUW711	T.DICOCCON CI9309/AE.SQUARROSA (409)/3/MILAN/S87230//BAV92/4/2*MILAN/S87230//BAV92	India	2019	Public
6	BHU-25	KIRITATI/4/2*SERI.1B*2/3/KAUZ*2/BOW//KAUZ/5/CMH81.530	India	2020	Public
7	BHU-31	QUAIU #1/SOLALA//QUAIU #2	India	2020	Public
8	Rajendra Gehun 02	T.DICOCCON CI9309/AE.SQUARROSA (409)//MUTUS/3/2*MUTUS	India	2021	Public
9	Zincol-16	OASIS/SKAUZ//4*BCN/3/2*PASTOR/4/T.SPELTA PI348449/5/BACEU #1/6/WBLL1*2/CHAPIO.	Pakistan	2016	Public
10	Akbar-19	BECARD/QUAIU#1	Pakistan	2020	Public
11	Nawb-21	HGO94.7.1.12/2*QUAIU #1/3/VILLA JUAREZ F2009/SOLALA//WBLL1*2/BRAMBLING	Pakistan	2021	Public
12	BARI-Gom 33	Kachu/Solala	Bangladesh	2017	Public
13	Iniaf-Okinawa	Kachu/Solala	Bolivia	2018	Public
14	Nohely F2018	T.DICOCCON CI9309/AE.SQUARROSA (409)//MUTUS/3/2*MUTUS	Mexico	2018	Public
15	ZINC GAHUN 1	MELON//FILIN/MILAN/3/FILIN/5/CROC_1/AE.SQUARROSA (444)/3/T.DICOCCON PI94625/AE.SQUARROSA (372)//3*PASTOR/4/T.DICOCCON PI94625/AE.SQUARROSA (372)//3*PASTOR/6/AMUR	Nepal	2020	Public
16	ZINC GAHUN 2	T.DICOCCON CI9309/AE.SQUARROSA (409)//MUTUS/3/2*MUTUS	Nepal	2020	Public
17	BHERI-GANGA	MELON//FILIN/MILAN/3/FILIN/5/CROC_1/AE.SQUARROSA (444)/3/T.DICOCCON PI94625/AE.SQUARROSA (372)//3*PASTOR/4/T.DICOCCON PI94625/AE.SQUARROSA (372)//3*PASTOR	Nepal	2020	Public
18	HIMA-GANGA	CHONTE*2/SOLALA//2*BAJ #1	Nepal	2020	Public
19	KHUMAL-SHAKTI	FRNCLN*2/7/CMH83.1020/HUITES/6/CMH79A.955/4/AGA/3/4*SN64/CNO67//INIA66/5/NAC/8/WBLL1*2/KURUKU//HEILO/9/WBLL1*2/KURUKU//HEILO	Nepal	2020	Public
20	Borlaug-2020	ROLF01/4/BOW/NKT//CBRD/3/CBRD/5/FRET2/TUKURU//FRET2	Nepal	2020	Public

We compared GZnC and GFeC measured in the same set of lines in different EYTBW 2021 environments and observed that the mean GZnC in the irrigated environment (43.3 ppm) was slightly higher than the mean concentration in the drought-stressed environment (39.8 ppm), whereas the mean GFeC in both the environments were similar (39.3 ppm). Our results are contrasting to previous reports of higher GZnC in drought-stressed environments (Genc et al., 2009; Velu et al., 2016b) but are in agreement with that of Magallanes-López et al. (2017), who reported significantly lower GZnC in the reduced irrigation environment. While dry conditions are expected to make the grain smaller and increase the GZnC (Genc et al., 2009; Velu et al., 2016b), we only used a moderately drought-stressed environment in this study, and hence, the concentrations might differ from those in severely drought-stressed environments. In addition, the correlations between GZnC and GFeC measured in irrigated and drought-stressed environments were 0.43 and 0.31, respectively, which is encouraging and points to the feasibility of indirect selection for especially GZnC in either of these environments and the development of biofortified varieties for low rainfall regions where zinc deficiency is prevalent (Genc et al., 2009).

We also observed that the GZnC and GFeC evaluated in the irrigated and small plots environments had moderately high correlations of 0.46 and 0.35, respectively, indicating the possibility of indirect selection in space-limiting small plots, which would be resource and space-efficient compared to full-size yield trial plots and facilitate screening a large number of lines before the yield trial stage. Moderate mean year-to-year correlations of 0.34 and 0.42 were observed for GZnC and GFeC, respectively, which is comparable to previous reports (Velu et al., 2017c; Alomari et al., 2018) and indicates a strong environmental effect on the traits. We also observed a positive and moderate correlation of 0.5 between GZnC and GFeC, indicating that simultaneous improvement for these traits is possible as also observed in previous studies (Velu et al., 2011, 2019; Xu et al., 2012; Guzmán et al., 2014; Srinivasa et al., 2014; Khokhar et al., 2020; Rathan et al., 2021). Analysis of population structure did not show clearly distinguishable clusters of lines from the BW and ZN improvement programs, which is expected given that several high-yielding BW lines with other preferred traits are used as parents in the ZN improvement program through a limited backcross approach (Velu et al., 2015).

We performed a large genome-wide association study for GZnC and GFeC using lines from CIMMYT’s BW and ZN improvement programs that were evaluated in multiple environments and years. Among the 1,207 markers that were significant in 73 datasets at a *p*-value threshold of 0.001, we observed that only 35% of them were significant in more than one dataset and the highest number of datasets in which a marker was significant was only seven. This could be because of the missing marker data in some datasets, the variable marker allele frequencies in different nurseries owing to the different parents used, and the effects of the environment on GZnC and GFeC (Pfeiffer and McClafferty, 2008; Velu et al., 2012; Guttieri et al., 2015; Crespo-Herrera et al., 2017). The 141 markers that were consistently associated with GZnC and GFeC in three or more datasets in this study were located on all wheat chromosomes except 3A and 7D, with the largest number of significant marker-trait associations on chromosome 7B (44), followed by chromosomes 6B (15), 1B (10), 7A (10), 1A (7), 5A (7), 5B (7), 2D (6), 4A (6), 4B (5), 2A (4), 2B (4), 6D (4), 1D (3), 3B (3), 4D (2), 5D (2), 3D (1), and 6A (1). The positions of these significant markers were compared with positions of previously reported markers for GZnC and GFeC that were either available on the RefSeq v1.0 or obtained from their sequences using the Basic Local Alignment Search Tool available in the Triticeae Toolbox (Blake et al., 2016).

On chromosome 1AL, we identified markers between 551428126 and 558541135 bps with high LD (Mean D′ = 0.99) that were associated with GFeC only and markers 1A_536664231 and 1A_570615890 that were associated with both GZnC and GFeC. Among them, marker 1A_536664231 was only 11.6 kbs away from *QGFe.co-1A* reported to be associated with GFeC (Liu et al., 2019). Markers 1A_551428126 and 1A_570615890 were in the same position as several GZnC and GFeC-associated markers between 551461626 (Ra_c5683_1762) and 571789571 (CAP12_c3758_112) bps reported by Cu et al. (2020) and are indicating the same locus. Furthermore, marker *IWA8135* reported to be associated with GZnC (Baranwal et al., 2022) was only 29.5 kbs away from the marker 1A_558541135 in our study, while GFeC associated markers AX-158569244 (542434057 bps) and AX-109301351 (543618767 bps) (Alomari et al., 2019) were flanked by the markers significant in this study and are all indicating the same locus.

On chromosome 1BL, we observed that, while marker 1B_634242993 was associated with GZnC only, several markers between 625077030 and 641131482 bps were also associated with GFeC only, and markers 1B_635977848 and 1B_641218839 were associated with both GZnC and GFeC. Among them, GZnC-associated marker 1B_634242993 was only 36.7 kb away from marker *wpt-1403* that flanked the GZnC QTL *QGZn.sar_1Btsk* (Velu et al., 2017c). Similarly, marker Excalibur_c66196_256 (625569783 bps) associated with GFeC (Cu et al., 2020) was flanked by GFeC associated markers 1B_625232015 and 1B_631257174 in this study and they indicate the same locus. On chromosome 2AL, we identified GZnC associated markers 2A_428372781 and 2A_760579448 and GFeC associated markers 2A_755740567 and 2A_770713812. Among them, markers 2A_755740567, 2A_760579448, and 2A_770713812 are in the same region as several GFeC-associated markers reported by Alomari et al. (2019) between 729175064 (AX-94482613) and 770007136 bps (AX-109961625).

On chromosome 2BL, markers 2B_602765846 and 2B_667426036 were associated with both GZnC and GFeC in this study and they flanked, (i) the GZnC region tagged by markers Excalibur_c19649_1500, Excalibur_rep_c67411_210, Excalibur_c11392_1193, and wsnp_Ex_c9729_16071358 (Velu et al., 2018) that were between 616955895 and 643684946 bps; (ii) the GZnC region tagged by markers GENE-1125_32 and Tdurum_contig54925_225 (Cu et al., 2020) that were between 637573847 and 637574357 bps; and (iii) the GZnC associated marker BS00012036_51 (Wang et al., 2021) that was at 646215529 bps. On chromosome 2DS, marker 2D_135478757 associated with both GZnC and GFeC in this study was 10.8 Mbs away from GZnC associated marker Kukri_c14902_1112 (Velu et al., 2018). On chromosome 3BL, markers 3B_756626946 and 3B_794884822 associated with GZnC and GFeC, respectively, in this study flanked GZnC associated marker IWB64607 (Baranwal et al., 2022) at 772399720 bps.

On chromosome 4AL, marker S4A_681683160 (Bhatta et al., 2018) reported to be associated with GZnC was flanked by markers 4A_558059830 and 4A_646730848 that were significantly associated with GZnC and GFeC in this study. In addition, GZnC associated marker Kukri_c25823_443 at 631922580 bps (Cu et al., 2020) was flanked by significant markers 4A_672877364 (GZnC) and 4A_703167907 (GZnC and GFeC). On chromosome 4BS, GFeC-associated marker 4B_21379808 was 9.5 Mb away from the *Rht*-B1 gene that has been previously associated with GZnC and GFeC (Velu et al., 2017b). On chromosome 5AS, marker 5A_6960731 associated with GFeC was 2.1 Mbps away from the GZnC associated marker wsnp_Ex_c16551_25060833 reported by Velu et al. (2018) and 2.9 Mbps away from the GFeC associated marker wsnp_Ex_c28908_37989320 reported by Cu et al. (2020). On chromosome 5AL, GZnC associated marker S5A_552354940 (Bhatta et al., 2018), GFeC associated QTL *QGFe.co-5A.1* and *QGFe.co-5A.2* (Liu et al., 2019), and GZnC associated marker *IWA2365* (Baranwal et al., 2022) were located in the interval tagged by GZnC and GFeC associated markers 5A_510211856, 5A_585608055, and 5A_618265845 and an LD block comprising markers 5A_607673246, 5A_608344326, and 5A_608360107 (Mean D′ = 0.1) that were significant in this study.

On chromosome 5BL, GFeC associated QTL *QGFe.cimmyt-5B_P1* (Crespo-Herrera et al., 2017) and several GZnC and GFeC associated markers between 517867135 (wsnp_RFL_Contig1570_778491) and 562970329 (wsnp_Ex_c13485_21225504) bps that were reported by Cu et al. (2020) were in the interval tagged by significant markers 5B_316011853, 5B_559726088, 5B_571635082, 5B_586610468, 5B_586715328, and 5B_592792409 that were associated either with GZnC only or with both GZnC and GFeC in the study. On chromosome 5DL, the GFeC-associated markers AX-158587148 and AX-158543037 (Alomari et al., 2019) were in the interval tagged by markers 5D_385132035 and 5D_433017840 that were associated with GZnC and GFeC, respectively, in this study. On chromosome 6AS, the GFeC associated marker 6A_61080142 was in the location of the GZnC QTL *QGZn.cimmyt-6A_P1* (Crespo-Herrera et al., 2017).

On chromosome 6B, several markers between 153561917 and 471154484 bps, some of which were in high LD with few others, were significantly associated with GZnC only in this study. Among them marker 6B_153561917 was 18.9 Mbps away from the *GPC*-B1 gene that has been previously reported to be associated with GZnC and GFeC (Uauy et al., 2006; Distelfeld et al., 2007; Velu et al., 2017a) and was also associated with grain and flour protein content (Juliana et al., 2019). In addition, GZnC associated QTL *QGZn.co-6B.2* (Liu et al., 2019) was flanked by markers 6B_174550372 and 6B_183278496 that were significant in this study.

On chromosome 7B, GZnC associated QTL/markers, (i) *QGZn.cimmyt-7B_1P2* between 485838522 and 506414028 bps (Crespo-Herrera et al., 2017) was flanked by the GZnC associated markers 7B_393314447 and 7B_516450006; (ii) *QGZn.cimmyt-7B_1P1* (Crespo-Herrera et al., 2017) was in the same position as the GZnC and GFeC associated markers 7B_121570273 and 7B_126740591; (iii) *QGZn.cimmyt-7B_2P1* (Crespo-Herrera et al., 2017) was in the same position as the GFeC associated markers 7B_131374909 and 7B_168075214 that were in high LD (D′ = 0.97); (iv) Tdurum_contig65979_289 (539220004 bps), a stable GZnC associated marker (Tong et al., 2022) was only 0.65 Mbps away from GZnC associated marker 7B_530585647 that was significant in this study. In addition, GZnC associated markers reported by Wang et al. (2021) GZnC between 182142433 bps and 190801271 bps were flanked by the GFeC associated markers 7B_168075214 and 7B_196275863 that were significant in this study and constituted an LD block (D′ = 1).

We have validated several previously reported QTL and markers associated with GZnC and GFeC, in addition to reporting many novel associations, which together provide important insights into the genetic basis of these micronutrients. We have also reported several markers that were significantly associated with GZnC and GFeC in more than one environment among the irrigated, water-limiting drought-stressed, and space-limiting small plots environments. These provide strong evidence for the shared genetic basis of these micronutrient concentrations in different environments and indicate the feasibility of indirect selection for GZnC and GFeC in either of the environments depending on the cost and resources (i.e., small plots might be cheaper and space-saving) and the target population of environments where the biofortified lines will be grown (i.e., if the target areas are prone to drought, direct selection for GZnC and GFeC in the drought-stressed environment is essential and it can favor indirect selection for the irrigated environment).

Several markers associated with both GZnC and GFeC have been identified in this study, which is in agreement with previous studies reporting overlapping genomic regions associated with these traits (Xu et al., 2012; Crespo-Herrera et al., 2016; Tiwari et al., 2016; Velu et al., 2017c) and reinstates the possibility of simultaneously improving them. Our results also indicated that the maximum additive effects of the GZnC and GFeC associated markers on the traits were only 1.7 ppm and 1.3 ppm, respectively, which taken together with the large number of marker-trait associations identified in this study suggest a quantitative genetic control of GZnC and GFeC by many loci with small effects as reported previously (Shi et al., 2008; Genc et al., 2009; Hao et al., 2014; Alomari et al., 2018). Overall, our findings provide key insights into the complex genetic basis of GZnC and GFeC in bread wheat and imply limited prospects for implementing marker-assisted selection. Hence, a genome-wide marker-based selection approach like genomic selection that facilitates selection on the additive effects of multiple loci might be more appropriate for increasing the selection accuracy, enriching favorable alleles, and subsequently accelerating genetic gains for these traits (Meuwissen et al., 2001; Heffner et al., 2009; Velu et al., 2016a).

## Data Availability Statement

The datasets presented in this study can be found in online repositories. The names of the repository/repositories and accession number(s) can be found in the article/Supplementary Material.

## Author Contributions

PJ designed the study, performed the analyses, and wrote the first draft of the manuscript. RS and VG designed the experiments, developed the lines, and supervised the phenotyping. LC-H, SM, and JH-E were involved in line development and trial management. JP and SS were involved in generating the genotyping data. All authors reviewed the manuscript and approved the submitted version.

## Conflict of Interest

The authors declare that the research was conducted in the absence of any commercial or financial relationships that could be construed as a potential conflict of interest.

## Publisher’s Note

All claims expressed in this article are solely those of the authors and do not necessarily represent those of their affiliated organizations, or those of the publisher, the editors and the reviewers. Any product that may be evaluated in this article, or claim that may be made by its manufacturer, is not guaranteed or endorsed by the publisher.

## Footnotes

1
http://visualization.ritchielab.org/phenograms/plot

## Supplementary Material

Supplementary Table 1 Grain zinc and iron concentrations for the 5,585 breeding lines from the International Maize and Wheat Improvement Centre’s bread wheat and zinc improvement programs. The best linear unbiased estimates (BLUEs) for grain zinc and iron concentrations in the yield trial bread wheat (YTBW), elite yield trial bread wheat (EYTBW), yield trial zinc (YTZN), and elite yield trial zinc (EYTZN) lines evaluated in the bed planting 5 irrigations environment (B5IR), bed planting 2 irrigations environment (B2IR) or small plots (SP) are given, along with the concentrations in the parcela chica (small plots) zinc (PCZN) lines.

Supplementary Table 2 Statistical analysis of grain zinc and iron concentration in the bread wheat and zinc lines expressed in parts per million. BLUEs, best linear unbiased estimates; YTBW, yield trial bread wheat; EYTBW, elite yield trial bread wheat; YTZN, yield trial zinc; EYTZN, elite yield trial zinc; PCZN, parcela chica (small plots) zinc; B5IR, bed planting 5 irrigations; B2IR, bed planting 2 irrigations; SP, small plots.

Supplementary Table 3 Pearson’s correlations between grain zinc and iron concentrations in different replications, environments, and years. Rep, Replication; BLUEs, best linear unbiased estimates; YTBW, yield trial bread wheat; EYTBW, elite yield trial bread wheat; YTZN, yield trial zinc; EYTZN, elite yield trial zinc; PCZN, parcela chica (small plots) zinc; B5IR, bed planting 5 irrigations; B2IR, bed planting 2 irrigations; SP, small plots.

Supplementary Table 4 The 1,207 markers that were significantly associated with grain zinc and iron concentrations at a *p*-value threshold of 0.001 and their significance after Bonferroni correction for multiple testing at an alpha level of 0.2. Rep, Replication; BLUEs, best linear unbiased estimates; YTBW, yield trial bread wheat; EYTBW, elite yield trial bread wheat; YTZN, yield trial zinc; EYTZN, elite yield trial zinc; PCZN, parcela chica (small plots) zinc; B5IR, bed planting 5 irrigations; B2IR, bed planting 2 irrigations; SP, small plots.

Supplementary Table 5 The 67 markers significantly associated with grain zinc concentration only in different datasets, with their *p*-values and the number of datasets that they were significantly associated in. Rep, Replication; BLUEs, best linear unbiased estimates; YTBW, yield trial bread wheat; EYTBW, elite yield trial bread wheat; YTZN, yield trial zinc; EYTZN, elite yield trial zinc; PCZN, parcela chica (small plots) zinc; B5IR, bed planting 5 irrigations; B2IR, bed planting 2 irrigations; SP, small plots.

Supplementary Table 6 Markers significantly associated with grain zinc concentration only in different datasets with the range in their *p*-values, additive effects, and percentage variation explained.

Supplementary Table 7 The 45 markers significantly associated with grain iron concentration only in different datasets, with their *p*-values and the number of datasets that they were significantly associated in. Rep, Replication; BLUEs, best linear unbiased estimates; YTBW, yield trial bread wheat; EYTBW, elite yield trial bread wheat; YTZN, yield trial zinc; EYTZN, elite yield trial zinc; PCZN, parcela chica (small plots) zinc; B5IR, bed planting 5 irrigations; B2IR, bed planting 2 irrigations; SP, small plots.

Supplementary Table 8 Markers significantly associated with grain iron concentration only in different datasets with the range in their *p*-values, additive effects, and percentage variation explained.

Supplementary Table 9 The 29 markers significantly associated with both grain zinc and iron concentrations in different datasets, with their *p*-values and the number of datasets that they were significantly associated in. Rep, Replication; BLUEs, best linear unbiased estimates; YTBW, yield trial bread wheat; EYTBW, elite yield trial bread wheat; YTZN, yield trial zinc; EYTZN, elite yield trial zinc; PCZN, parcela chica (small plots) zinc; B5IR, bed planting 5 irrigations; B2IR, bed planting 2 irrigations; SP, small plots.

Supplementary Table 10 Markers significantly associated with both grain zinc and iron concentration in different datasets with the range in their *p*-values, additive effects, and percentage variation explained.
