# Supplementary material for: Genome-Wide Association Mapping Identifies Key Genomic Regions for Grain Zinc and Iron Biofortification in Bread Wheat
Source: Front Plant Sci. 2022 Jun 30;13:903819. doi: 10.3389/fpls.2022.903819 (PMC9280339; doi:10.3389/fpls.2022.903819)

**Linkage disequilibrium between the significant markers on chromosome 1A. The standardized disequilibrium coefficients (D’) are shown in the lower-left matrix and correlations between alleles at the two marker loci (r^2^) are shown in the upper-right matrix.**
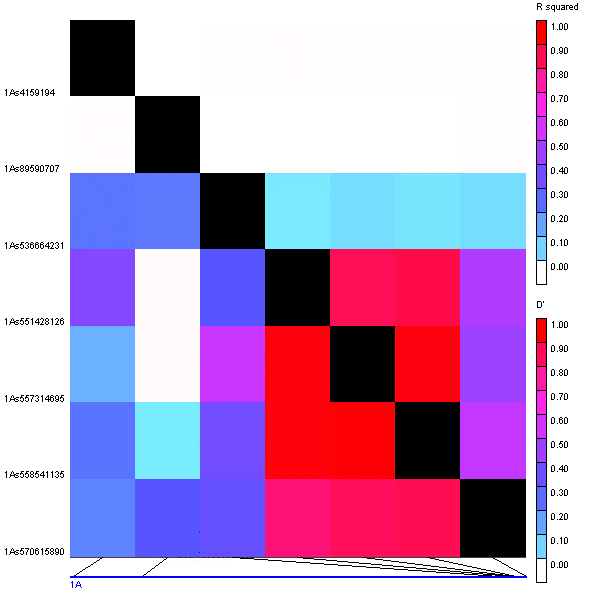


**Linkage disequilibrium between the significant markers on chromosome 1B. The standardized disequilibrium coefficients (D’) are shown in the lower-left matrix and correlations between alleles at the two marker loci (r^2^) are shown in the upper-right matrix.**
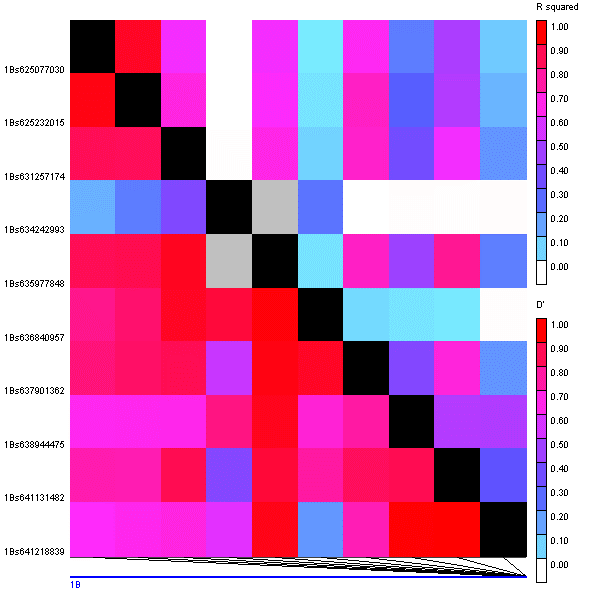


**Linkage disequilibrium between the significant markers on chromosome 1D. The standardized disequilibrium coefficients (D’) are shown in the lower-left matrix and correlations between alleles at the two marker loci (r^2^) are shown in the upper-right matrix.**
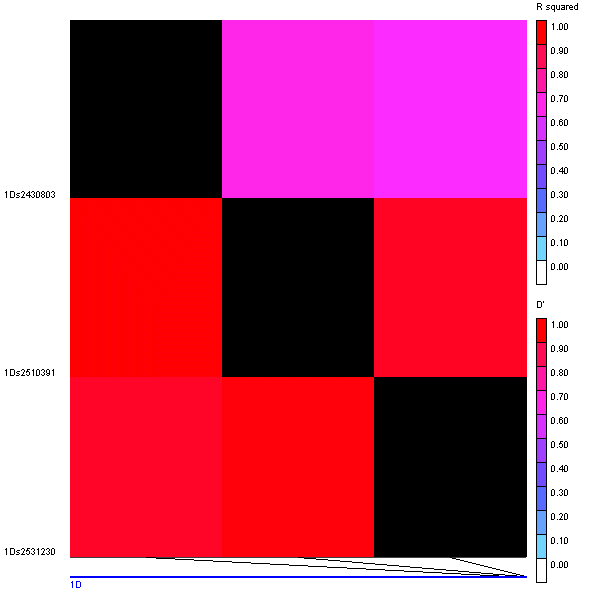


**Linkage disequilibrium between the significant markers on chromosome 2A. The standardized disequilibrium coefficients (D’) are shown in the lower-left matrix and correlations between alleles at the two marker loci (r^2^) are shown in the upper-right matrix.**
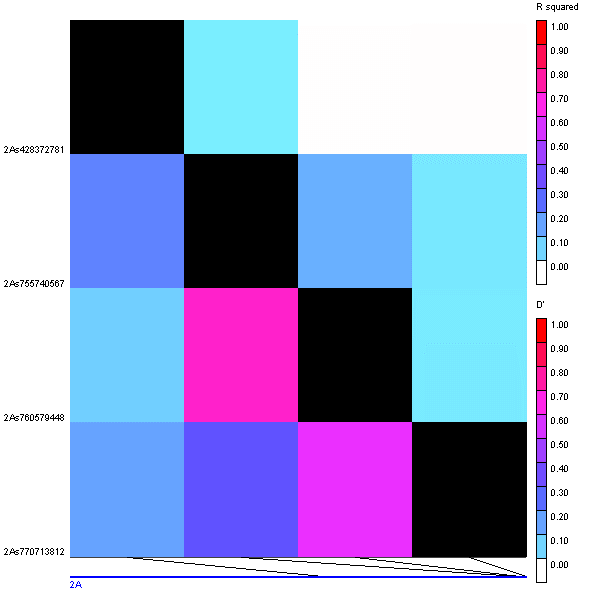


**Linkage disequilibrium between the significant markers on chromosome 2B. The standardized disequilibrium coefficients (D’) are shown in the lower-left matrix and correlations between alleles at the two marker loci (r^2^) are shown in the upper-right matrix.**
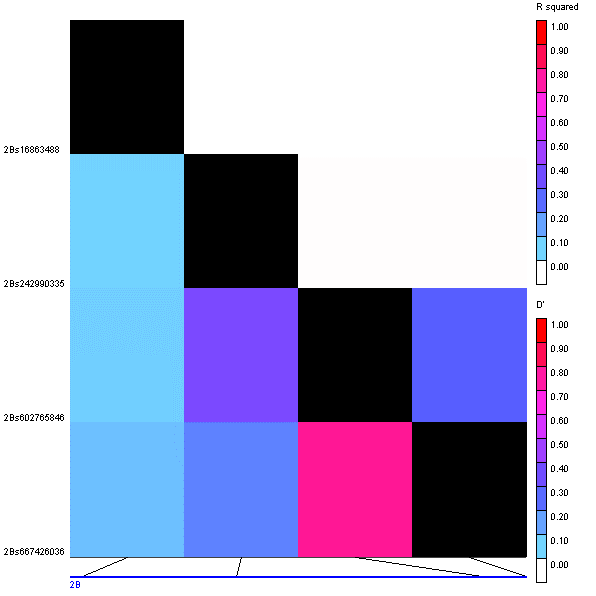


**Linkage disequilibrium between the significant markers on chromosome 2D. The standardized disequilibrium coefficients (D’) are shown in the lower-left matrix and correlations between alleles at the two marker loci (r^2^) are shown in the upper-right matrix.**
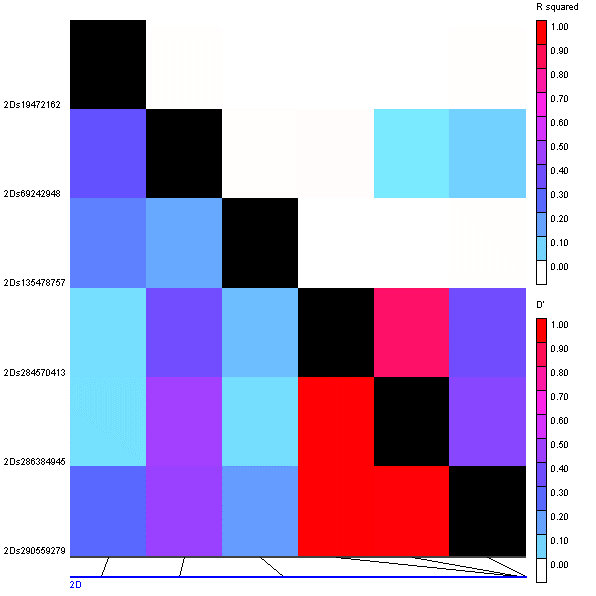


**Linkage disequilibrium between the significant markers on chromosome 3B. The standardized disequilibrium coefficients (D’) are shown in the lower-left matrix and correlations between alleles at the two marker loci (r^2^) are shown in the upper-right matrix.**
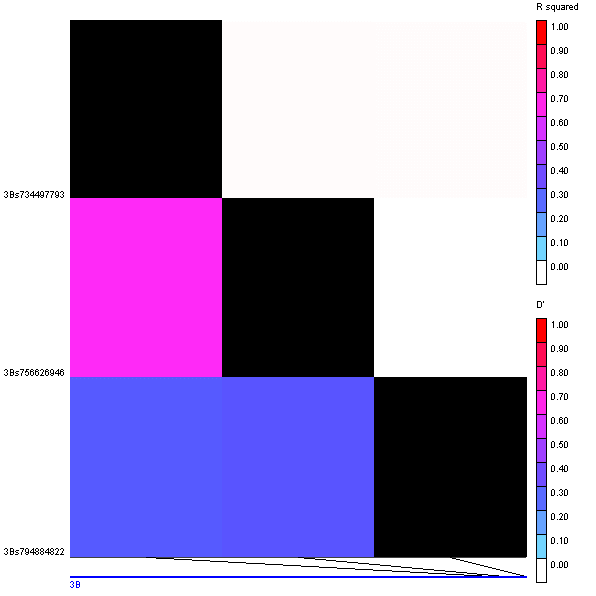


**Linkage disequilibrium between the significant markers on chromosome 4A. The standardized disequilibrium coefficients (D’) are shown in the lower-left matrix and correlations between alleles at the two marker loci (r^2^) are shown in the upper-right matrix.**
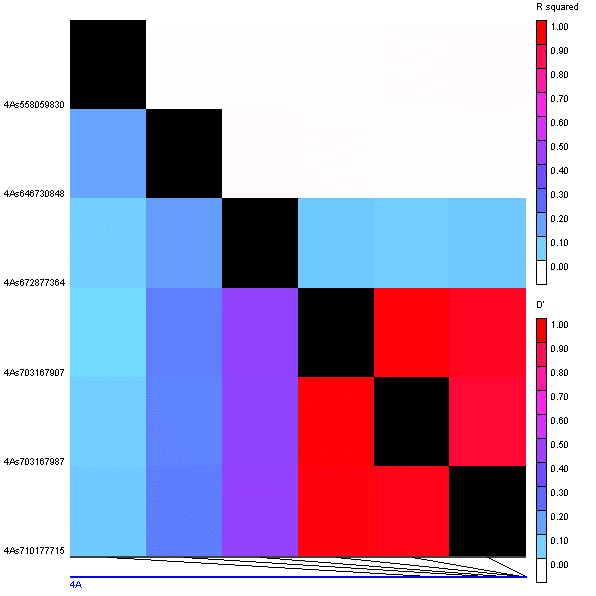


**Linkage disequilibrium between the significant markers on chromosome 4B. The standardized disequilibrium coefficients (D’) are shown in the lower-left matrix and correlations between alleles at the two marker loci (r^2^) are shown in the upper-right matrix.**
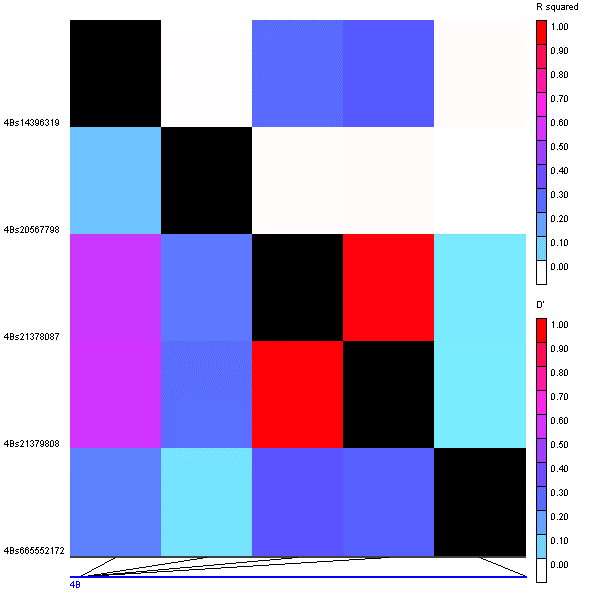


**Linkage disequilibrium between the significant markers on chromosome 4D. The standardized disequilibrium coefficients (D’) are shown in the lower-left matrix and correlations between alleles at the two marker loci (r^2^) are shown in the upper-right matrix.**
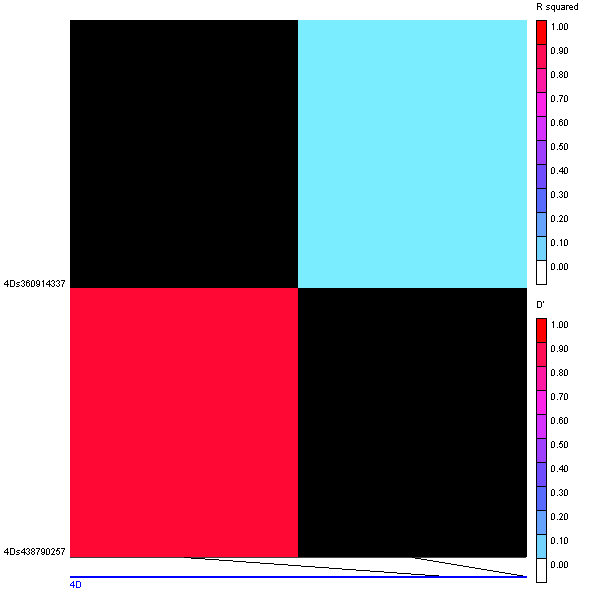


**Linkage disequilibrium between the significant markers on chromosome 5A. The standardized disequilibrium coefficients (D’) are shown in the lower-left matrix and correlations between alleles at the two marker loci (r^2^) are shown in the upper-right matrix.**
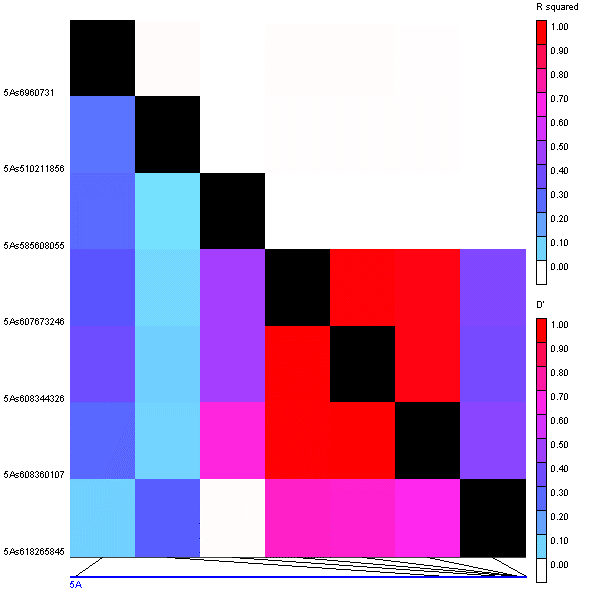


**Linkage disequilibrium between the significant markers on chromosome 5B. The standardized disequilibrium coefficients (D’) are shown in the lower-left matrix and correlations between alleles at the two marker loci (r^2^) are shown in the upper-right matrix.**
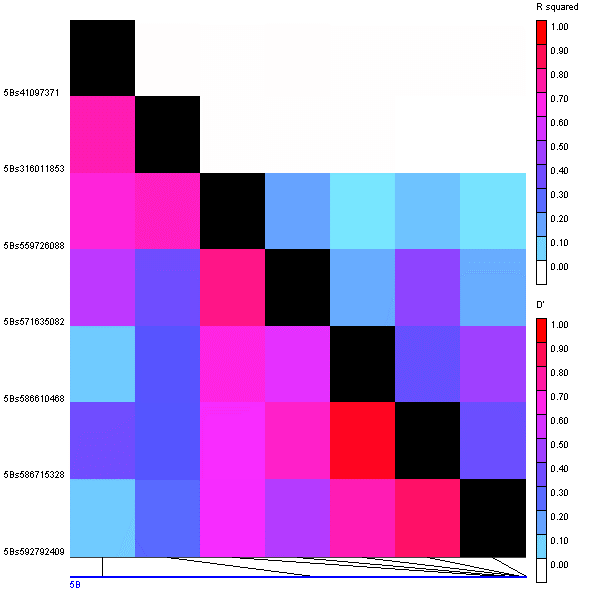


**Linkage disequilibrium between the significant markers on chromosome 5D. The standardized disequilibrium coefficients (D’) are shown in the lower-left matrix and correlations between alleles at the two marker loci (r^2^) are shown in the upper-right matrix.**
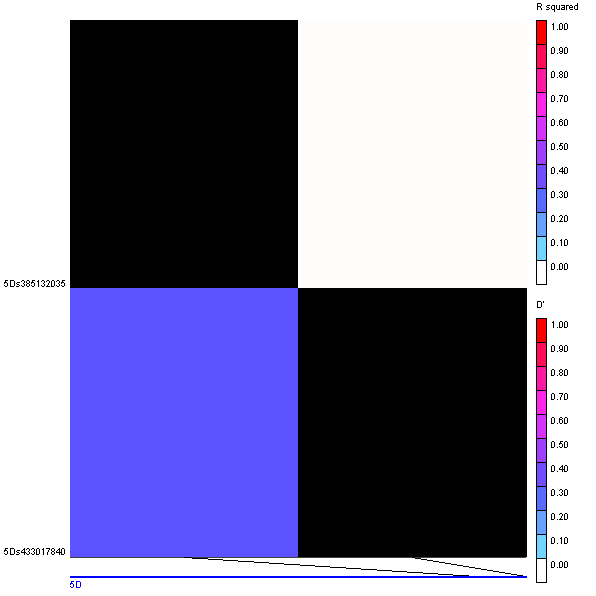


**Linkage disequilibrium between the significant markers on chromosome 6B. The standardized disequilibrium coefficients (D’) are shown in the lower-left matrix and correlations between alleles at the two marker loci (r^2^) are shown in the upper-right matrix.**
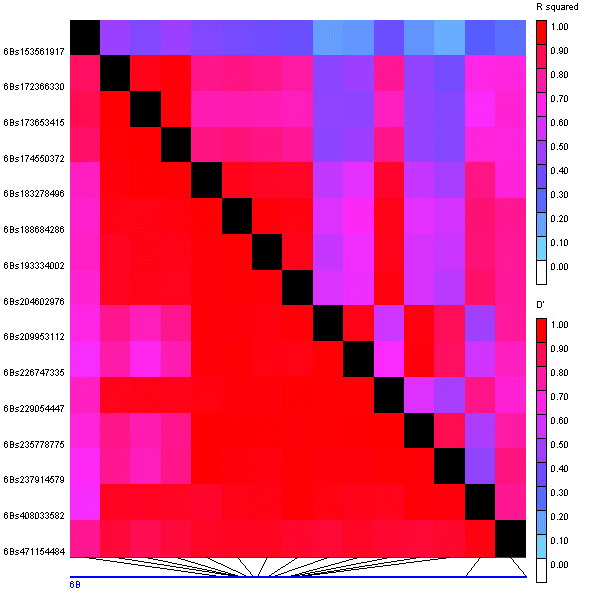


**Linkage disequilibrium between the significant markers on chromosome 6D. The standardized disequilibrium coefficients (D’) are shown in the lower-left matrix and correlations between alleles at the two marker loci (r^2^) are shown in the upper-right matrix.**
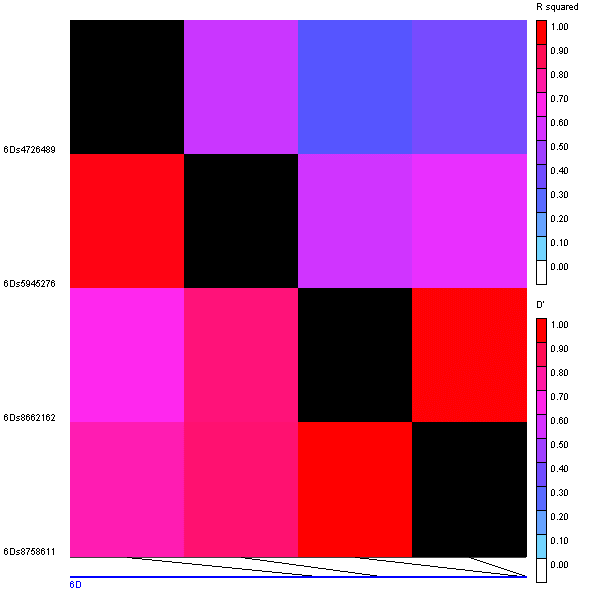


**Linkage disequilibrium between the significant markers on chromosome 7A. The standardized disequilibrium coefficients (D’) are shown in the lower-left matrix and correlations between alleles at the two marker loci (r^2^) are shown in the upper-right matrix.**
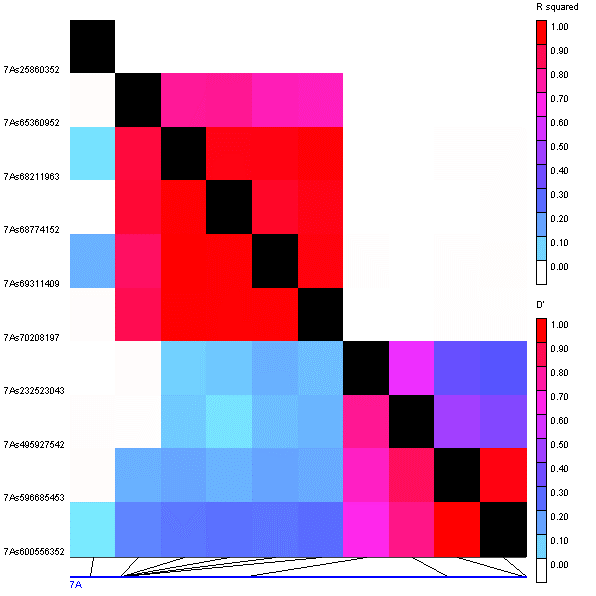


**Linkage disequilibrium between the significant markers on chromosome 7B. The standardized disequilibrium coefficients (D’) are shown in the lower-left matrix and correlations between alleles at the two marker loci (r^2^) are shown in the upper-right matrix.**
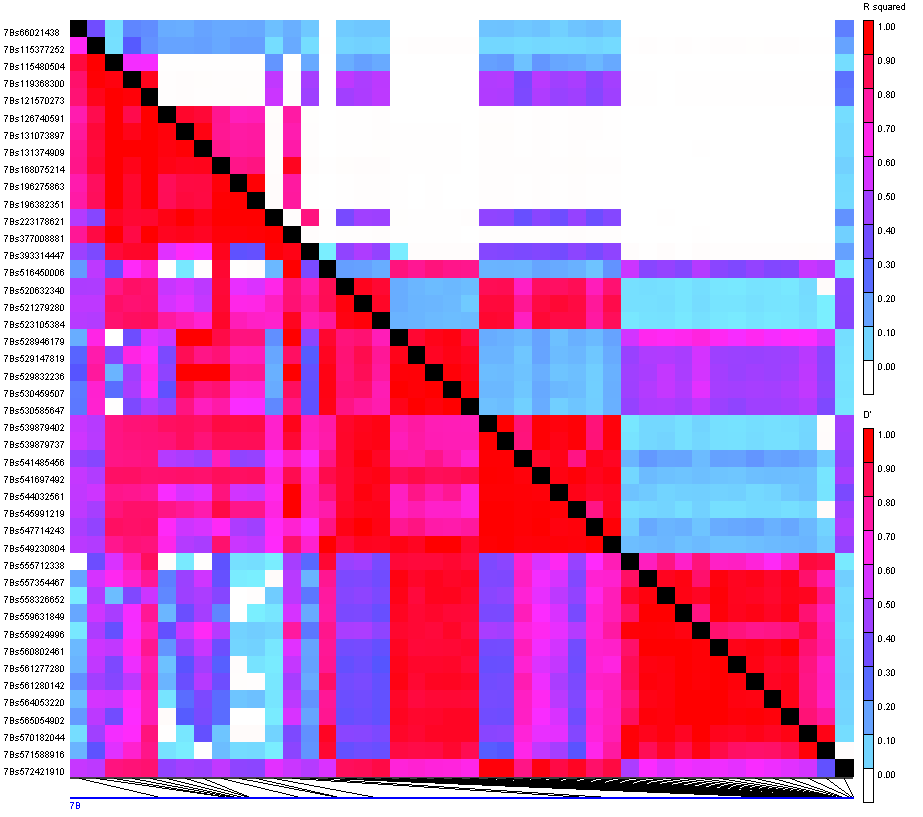

Supplement: Supplementary file 1 [file Data_Sheet_2.docx]
